# Medical negligence in the age of statistically superior AI

**DOI:** 10.1093/medlaw/fwag007

**Published:** 2026-03-18

**Authors:** Amelie S Berz

**Affiliations:** Faculty of Law, University of Oxford, St Cross Building, St Cross Road, Oxford OX1 3UL, United Kingdom

## Abstract

As artificial intelligence (AI) systems increasingly outperform human clinicians in specific diagnostic tasks, legal debates have turned to whether such statistical superiority should create new obligations in medical practice. This article proposes a two-stage transparency framework, distinguishing ‘pre-deployment transparency’ from ‘post-deployment interpretability’, to clarify when clinicians may, must, or must not use or rely upon AI systems. It argues that duties to adopt or rely on AI arise only where institutional endorsement and meaningful transparency enable doctors to make informed, context-sensitive judgments. Legal responsibility in AI-assisted care must rest on institutional validation and explainability, not on statistical performance alone. The article further shows that, consistent with existing case law, courts may draw adverse inferences from evidentiary gaps created by AI opacity, particularly when a party fails to preserve or disclose information within its control. This framework preserves clinical judgment and patient trust while ensuring that overall statistical gains do not mask systematic harms to minority groups. It concludes with recommendations for adapting medico-legal standards to the growing role of AI without displacing the clinician’s role as the legally accountable decision-maker.

## I. Introduction

The increasing integration of artificial intelligence (AI) into clinical decision-making has reignited foundational questions in the law of medical negligence. As AI-driven medical tools begin to exceed human capabilities in tasks such as parsing complex, multisystem data^1^ or detecting early-stage cancers,^2^ and as the UK government pursues its ambition to make the National Health Service (NHS) ‘the most AI-enabled care system in the world’,^3^ a familiar legal question resurfaces with new technological urgency: does a physician breach the duty of care by *declining to* defer to a machine whose statistical performance outstrips the average physician?^4^ This article argues that, even in cases where the machine’s outputs surpass human judgment, negligence liability does not automatically follow from a clinician’s failure to defer. This is not only because of persistent uncertainties about the machine’s reliability in real-world clinical settings, but more fundamentally because the legal standard of reasonableness cannot be collapsed into statistical superiority. A clinician’s obligations turn on what it is reasonable for a human decision-maker to grasp and act on in the circumstances—particularly where many AI systems remain opaque in operation and constrained in use.

Two core questions frame this article’s analysis. First, under what conditions does the duty of care permit, and ultimately *require*, a clinician to use an AI-enabled diagnostic tool? Before we can say when a doctor must use such a tool, we must be clear when it is even legally permissible to do so. Secondly, once an AI system is in use, how should the doctor’s duty of care guide their engagement with its diagnoses, predictions, or recommendations? The article proposes a two-stage transparency framework. It distinguishes between the information that must be available before a tool is adopted (‘pre-deployment transparency’) and the interpretive resources that must accompany individual outputs (‘post-deployment interpretability’). On this account, duties to use or follow AI do not arise from statistical performance alone, but from a combination of institutional validation and meaningful transparency. The effect is to shift part of the justificatory burden away from individual clinicians and towards those responsible for designing, approving, and endorsing these systems, while preserving clinical responsibility at the point of care.

The analysis also addresses the evidentiary implications of algorithmic opacity. Where those who design or deploy AI systems are best placed to preserve and explain relevant information, evidentiary principles allow courts to draw appropriate inferences from failures of documentation or disclosure. Opacity should not improve the defendant’s forensic position where the relevant information lay within its control.

While policymakers and ethicists have examined AI’s regulatory and moral dimensions, the doctrinal implications for medical negligence remain underdeveloped. Early scholarship on civil liability for AI tended to treat such systems as functional substitutes for human actors, naturally directing attention towards product liability and the regulatory obligations of developers.^5^ Instruments like the EU AI Act partially continue in this vein, imposing safety and transparency duties on AI providers before their tools reach the market.^6^ But this ‘replacement’ model falters in the face of most contemporary AI systems, particularly in medicine, where the technology is better understood as augmenting rather than supplanting human decision-making. Even highly advanced systems provide outputs that remain subject to professional assessment and decision-making.^7^ Even where the now-withdrawn AI Liability Directive (AILD) moved beyond traditional product liability, it asserted that cases in which a clinician acts on AI-generated advice raise no distinctive difficulties for liability when compared with conventional negligence claims.^8^ This assertion glosses over substantial normative and practical complications.^9^ AI-specific vulnerabilities, such as confounding bias, spurious correlations, and inscrutable statistical pathways, undermine the straightforward assumption that clinicians can be expected to understand, interrogate, and take responsibility for the AI’s recommendations.^10^ In such conditions, even the conceptual underpinnings of vicarious liability begin to fray. AI systems are not employees; they are proprietary tools, designed and controlled by distant corporate actors.^11^ If accountability is not clarified, effective decision-making authority may shift from clinicians to opaque technical systems shaped by design choices and commercial incentives rather than professional obligations.^12^ That, of course, raises questions not only of negligence but of institutional governance.

This article proceeds in seven parts. Section II sets out the foundations of medical negligence in English tort law, focusing on the Bolam–Bolitho framework and its limits. Section III explains the nature of AI diagnostic systems, highlighting both their potential and the challenges of opacity, bias, and regulatory oversight. Section IV identifies the legal and ethical preconditions for lawful clinical use, including regulatory approval and informed consent. Section V addresses the problem of epistemic uncertainty and argues that statistical superiority alone cannot ground a duty to adopt AI. Section VI develops a process-based framework in which institutional endorsement and minimum standards of transparency structure when doctors may, and must, use AI tools. Section VII examines liability questions arising from disregarding or relying on AI predictions, with particular attention to record-keeping and evidentiary burdens. Section VIII considers how conflicts between human judgment and machine outputs might be resolved procedurally, before the article concludes by considering how negligence law should respond to the expanding role of AI in clinical practice.

## II. The legal framework: medical negligence

Liability in negligence is not imposed for making the wrong choice per se, but for failing to make a reasonable one. The standard of care^13^ under English tort law is objective. One is negligent when one’s conduct fails to conform to the level of care that a reasonably competent person undertaking the activity would exercise.^14^ By holding individuals to a common standard, the law enables us to rely—without inquiry into individual idiosyncrasies—on a baseline of reasonable conduct from those around us.^15^ It thereby facilitates both interpersonal trust and legal accountability. At the same time, it spares the courts the impossible burden of tailoring duties of care to the psychological profile or individual experience level of each defendant.^16^ Subjective elements that deviate from the objective standard are exceptions, for example, for children^17^ and the disabled.^18^ On the other side of the spectrum, relevant knowledge that goes above and beyond the ordinary will be attributed to the defendant.^19^

Where situations involve a special skill or competence, the test of whether there has been negligence is a comparison to the ordinary skilled person exercising the profession or skill. Therefore, any physician must meet the standard of care of a doctor ‘skilled in that particular art’.^20^ To assess the standard of care in medical negligence, the leading case *Bolam v Friern Hospital Management Committee*^21^ set out in 1957 that a doctor will not be considered negligent where they acted in accordance with a practice that is accepted as proper by a responsible body of medical opinion. The plaintiff had undergone electro-convulsive therapy without administering relaxant drugs or manual restraint to control the convulsive movements and suffered severe physical injuries. Due to the variety of acceptable medical practices at the time, the defendants were not found negligent. This principle was confirmed by the House of Lords in *Maynard v West Midlands Regional HA*.^22^

*Bolitho v City and Hackney HA*^23^ further specified the test in 1997 by establishing that the practice accepted as proper by the body of professionals must be based on logical and defensible grounds. If the professional opinion were illogical, a respective action would still be a breach of duty. In the specific case, the court ultimately held that the defendant was not negligent, as the failure to intubate the child, which led to its death, was based on a reasonable professional judgment. Lord Browne-Wilkinson suggested that the court did not see *Bolitho* as an amendment, but rather as a specification of the *Bolam* test since the ‘logic’-requirement had already been indicated in the demand for a ‘*responsible*’ body of opinion.^24^

The combination of the two tests avoids a commonly identified problem: custom rules facilitate fact-finding, shield defendants from a judge’s biases, and make tort adjudication more consistent and predictable. Yet, if the standard of care is only defined by custom, then tort liability might prevent socially beneficial changes and advancements. Any physician who innovates would risk liability in case of damage, even if the innovation on average is beneficial, while users of the customary method can successfully fend off all charges.^25^ The *Bolitho* test allows for the argument that a medical consensus and expert viewpoint may be wrong or outdated, for example, due to new research or improved technology. Courts have been prepared, under *Bolitho*, to scrutinize the non-use of available diagnostic technology where the small likelihood of serious harm rendered non-referral logically indefensible.^26^

Several considerations structures the general standard of care in negligence law: the magnitude of the risk, the seriousness of the potential harm, the burden of taking precautions, and the broader social utility of the defendant’s conduct. These factors form what is often termed the ‘negligence calculus’—not a formula, but a framework for reasoned judgment.^27^ Although the *Bolam* and *Bolitho* tests were not born of the negligence calculus in any explicit sense, they operate squarely within its terrain. As *Lord Browne-Wilkinson* lays out, ‘In particular, where there are questions of assessment of the relative risks and benefits of adopting a particular medical practice, a reasonable view necessarily presupposes that the *relative risks and benefits have been weighed* by the experts in forming their opinions.’^28^ While law does not require that the risk be quantified to the third decimal point, it declines to impose liability where risk was genuinely beyond contemporary awareness, even where the ultimate harm falls within the broader category of injury.^29^ *Bolam* provides courts with a principled proxy for the standard of care in fields where lay judgment cannot stretch; by deferring to responsible bodies of medical opinion, it translates the negligence calculus into professional terms. *Bolitho*, for its part, reasserts the court’s supervisory role.

In disclosing risks to a patient, a doctor must weigh the likelihood of those risks materialising and inform the patient about the nature, risks, benefits, and alternatives of a proposed treatment.^30^ Here, the doctor’s duty is not to act on the patient’s behalf, but to enable the patient to act for themselves. Informed consent is rooted in respect for autonomy and self-determination^31^: it is only valid if it is both informed and voluntary.^32^ Good medical practice^33^ in line with ethics and law requires that patients be supported in making informed choices, with emphasis on material risks^34^—those that a reasonable person would consider significant. Where diagnosis or outcomes are uncertain, this too must be explained.^35^ Failure to disclose a material risk that would have led the patient to choose differently may amount to negligence.^36^

As in all negligence cases, the burden of proof rests with the claimant, who must establish both breach and causation on the balance of probabilities.^37^ Medical negligence claims, however, often founder on evidential difficulties. The complexity of clinical decision-making, coupled with the asymmetry of information between doctor and patient, makes proof elusive. Although courts have, in the past, shifted the burden to defendants—particularly where hospitals hold superior knowledge—this approach has since been curtailed.^38^ Instead, judges increasingly rely on *clinical guidelines*, such as those issued by the National Institute for Health and Care Excellence (NICE), as evidentiary anchors. A physician who follows such guidance will ordinarily have strong evidence of reasonable practice, unless the guidelines themselves are demonstrably flawed.^39^ Conversely, a departure from established protocols invites scrutiny and calls for an ‘explanation’ to avoid an inference of negligence.^40^ Therefore, if a clinician departs without explanation, courts may find a breach of duty.

## III. AI in medicine: opportunities and challenges

*Artificial Intelligence* refers to computational systems capable of performing tasks associated with human cognition, such as learning, reasoning, and decision-making.^41^ This article focuses on advanced *analytical* machine learning (ML)^42^ models used in medical decision-support: systems that detect patterns in complex datasets and offer clinical insights based on statistical findings. It does not address interactive or generative AI models, like GPT-based report summarization, nor robotic tools requiring surgical supervision. Unlike traditional rule-based systems,^43^ these models operate as ‘black boxes’, offering little interpretive insight into how outputs are generated. While they can enhance diagnostic precision and streamline workflows, they lack the capacity for ethical reasoning and contextual judgment central to clinical care^44^ and can be viewed not as replacements but as cognitive augmentations.

AI deployment within the NHS is no longer experimental. ML systems are now used to support image-based diagnosis and screening, including AI-assisted analysis of prostate MRI scans, lung cancer detection pilots, and rapid stroke imaging assessment.^45^ Empirical evidence of performance gains is mounting. In a recent *Max Planck* study analysing over 40,000 diagnoses across 2100 realistic clinical vignettes, AI collectives exceeded the accuracy of 85 per cent of doctors. Groups combining humans and AI achieved significantly higher diagnostic accuracy than either alone, since their different error patterns complemented one another.^46^ Yet, AI’s clinical efficacy remains limited by inconsistent validation,^47^ data bias,^48^ and opacity.^49^ Designed, in part, to derive insights beyond human capability (‘unpredictability by design’ or ‘emergent behaviour’^50^), these systems often resist human comprehension. Efforts to render them interpretable—via ‘Explainable AI’ (XAI) techniques^51^ such as feature importance metrics providing either global insights into model behaviour or local explanations of specific decisions^52^—are crucial for both clinical trust and legal scrutiny.

The regulatory landscape includes the UK Medical Device Regulation 2002 (MDR),^53^ and the Data Protection Act 2018 domestically^54^; within the EU, the GDPR,^55^ the Regulation (EU) 2017/745 (EU MDR),^56^ and, from 2026, the EU AI Act,^57^ all of which govern the development, deployment, and data use of AI systems. According to Article 6(1), Annex I Section A no 11 of the EU AI Act, medical devices will be considered ‘high-risk’ where they are subject to third-party conformity assessment under the MDR. Articles 13(1) and (2), 14(1) and (4) of the AI Act impose transparency obligations on developers, yet these rules often exclude users and clinicians, raising concerns about the disempowerment of both doctors and patients.^58^ Systems must function reliably under foreseeable conditions, including human error and environmental noise.

## IV. Preconditions for the lawful use of AI in clinical practice

A foundational legal precondition to imposing a duty to use AI is that its use be lawful. A clinician cannot be required to use a device whose deployment itself would breach the standard of care. In both UK and EU law, this entails regulatory approval (via CE or UKCA marking)^59^ and valid informed consent. Under the UK MDR 2002 and EU MDR, most diagnostic AI tools fall into Class IIa or IIb, requiring notified body review.^60^ Use of an unapproved device may not only breach regulatory obligations but also constitute strong evidence of breach of duty in negligence^61^—effectively placing the burden of risk on the clinician.

In the EU, prior to market placement, Article 11 of the AI Act requires providers of high-risk AI systems to prepare comprehensive technical documentation, including an explanation of the system’s overall logic, the algorithms employed, key design decisions, and principal classification choices (Annex IV(2)(b)).

Informed consent adds a second dimension to lawful use. Patients are not expected to comprehend the technical intricacies of AI systems—any more than they are expected to understand the pharmacodynamics of a drug^62^—but they are entitled to be informed when such systems play a meaningful role in diagnosis or treatment. This is especially true where AI substantially supplants professional judgment, introduces novel risks through its opacity, or serves to compensate for deficiencies in expertise.^63^ Where it is known that an AI system performs less reliably in a defined patient subgroup, and the patient falls within that group, the resulting differential risk of misdiagnosis may amount to a material risk requiring disclosure under *Montgomery*. The position may be different where subgroup validation is merely absent, but no performance deficit is known; the duty to disclose is triggered not by abstract uncertainty, but by clinically significant and patient-specific risk.

Where none of the non-consent bases under GDPR Article 6(1)(b)–(f) apply (eg necessity for public interest), patient consent under Article 6(1)(a) is required for processing personal data beyond the immediate care context; for example, in cloud-based model training or third-party evaluation. The GMC’s guidance reinforces the need for context-sensitive communication, calibrated to the severity of the condition and the complexity of the decision involved.^64^ The challenge lies in balancing sufficient disclosure to preserve autonomy with the need to avoid informational overload. Excessive detail regarding model architecture or error margins may obscure rather than clarify decision-making.

In sum, the lawful deployment of AI in medicine rests on three interdependent pillars: regulatory approval, informed patient consent, and sound clinical judgment. Together, these constraints ensure that technological assistance does not displace professional accountability or erode the patient’s capacity to make informed choices.

## V. Epistemic uncertainty, probabilistic evidence, and the limits of a duty to use AI

A physician may, in principle, face liability for not using a high-performing AI-enabled device if, on the balance of probabilities, it would have avoided a misdiagnosis. This is not a matter of tortious ‘omission’ and the respective doctrine, but a question of the quality of conduct within a continuous process of care.^65^ The breach question turns on whether the overall decision-making fell short of what could reasonably be expected, given the circumstances. That judgment must take seriously the practical realities of AI implementation.

AI integration involves significant infrastructural commitments: system acquisition, regulatory compliance, staff training, and compatibility with clinical workflows. The marginal cost of using AI in practice,^66^ as opposed to its potential utility, remains difficult to quantify. The NHS, for example, has identified no fewer than 29 compliance steps for AI deployment, 20 of which are deemed legally necessary.^67^ Without clarity on reimbursement mechanisms and data governance, doctors may hesitate to adopt AI, even where tools are CE-marked and high-performing.

Beyond questions of cost and efficiency, a more fundamental barrier to the clinical integration of AI lies in the epistemic opacity and real-world unreliability of these systems. Empirical studies have revealed that widely used AI models in hospital settings have failed to detect up to two-thirds of critical injuries,^68^ underscoring the potential for serious harm when such tools are deployed in complex, high-stakes environments. The root cause often lies in a mismatch between training data and clinical reality: many models are developed using historical electronic health records, without a robust grasp of underlying medical physiology. As a result, they perform poorly when confronted with atypical patient presentations, such as abnormal vital signs. Even technically advanced architectures, including Long Short-Term Memory networks and Transformer-based models, have shown significant performance degradation under stress testing.^69^ While these systems may achieve high statistical indicators, such as a favourable Area Under the Receiver Operating Characteristic curve, such metrics are not, in themselves, proxies for safety or clinical trustworthiness. Unsurprisingly, clinicians often hesitate to defer entirely to algorithmic outputs, particularly in conditions of uncertainty or moral complexity. Human judgment remains the preferred point of recourse, even when AI models demonstrate superior aggregate accuracy, precisely because humans are capable of exercising moral discretion, contextual understanding, and accountability—capacities that machines, as yet, do not possess.^70^ Human decision-making is also perceived as more transparent than algorithmic reasoning, which may make clinicians more reluctant to rely on AI systems than on human judgment.^71^ Paradoxically, however, when AI is used not for decision delegation but for decision support, a reverse tendency emerges: clinicians may over-rely on algorithmic recommendations, becoming less vigilant in reviewing or correcting automated errors.^72^ Crucially, the field still lacks robust predictive frameworks for identifying when and how these models are likely to fail.

The task of anticipating and managing the risks associated with AI-enabled clinical decision-making is epistemically demanding in ways that differ from more familiar medical technologies. Two aspects of this challenge stand out: first, the difficulty in establishing the *absolute risk*—the likelihood that the AI system will produce an erroneous output; and secondly, the difficulty in assessing the *marginal risk*—the degree to which reliance on AI improves or worsens the outcome relative to human judgment. Both difficulties arise not from technological immaturity alone, but from the opacity and epistemic inaccessibility of AI systems as presently constructed.

On the first front, while AI users may be presented with performance metrics derived from training and validation data, these often mask more than they reveal. Current regulatory standards, such as CE or UKCA marking, fall short of offering the kind of epistemic warrant that clinicians take for granted in pharmaceutical regulation. A physician may not know the fine-grained details of a drug trial, but can rely on the fact that it met clearly articulated standards of safety and efficacy.^73^ No such assurance follows from existing AI validation regimes. Current AI standards are underdeveloped compared to those in other sectors.^74^ These models are trained on vast troves of historical data, sometimes numbering in the hundreds of billions of data points^75^; yet clinicians are rarely in a position to assess the relevance, quality, or representativeness of those datasets to their own patient populations. Hidden within such data may be structural biases, regional disparities, or unrecognized confounders that subtly distort the model’s reliability in practice. As in other instances, mathematical probability may guide policymakers in designing systemic rules, but offers little assistance in determining the odds of individual cases.^76^

This uncertainty resonates with a long-standing tension in legal theory: the distinction between population-level probabilities and case-specific proof. In *XYZ Ltd v Schering Health Care Ltd*,^77^ the court grappled with whether an epidemiological association between oral contraceptives and cardiovascular injury sufficed to establish causation under the ‘but for’ test. Faced with the evidentiary limitations of multifactorial causation, the court contemplated whether a ‘doubling of the risk’ standard might serve as an alternative. Such a move requires sound, complete data.^78^ This tension mirrors the AI context, where risk projections often rely on data too abstract or heterogeneous to be probative in individual cases. As scholars in evidence law have long pointed out,^79^ adjudication requires more than statistical probability. It requires interpretive judgment about agency, context, and individual entitlement precisely to avoid violating a person’s fundamental right to live free from threats, dangers, and harm.^80^ Hence, the persistent judicial reluctance to accept probabilistic causation in lieu of direct proof, however elusive. As judicial commentary has made clear, ‘[s]o long as medical science is unable to demonstrate, as a matter of fact, the aetiology of mesothelioma, data relating incidence to exposure is not a satisfactory basis for making findings of causation.’^81^ In other words, the enduring absence of definitive mechanistic understanding renders such data evidentially incomplete, both in law and in science.

Courts have turned to tools such as Bradford Hill’s causation criteria^82^—including strength of association, consistency, and biological plausibility—to complement statistical evidence with substantive explanatory frameworks.^83^ So too in the AI context: validation procedures must not only yield favourable metrics, but also furnish intelligible, context-sensitive rationales that allow clinicians—and courts—to assess the probability that a model’s use will, or will not, cause harm in a given case.

The second difficulty concerns the *marginal*, or *comparative*, risk^84^ posed by declining to use an AI system; that is, whether foregoing AI support amounts to falling below the standard of care. Unlike traditional technologies, AI systems purport not merely to assist, but to *outperform* human cognitive faculties in specific domains, such as radiological pattern recognition or diagnostic triage. Yet for that claim to carry legal weight, it must be benchmarked not against a general standard, but against the specific clinician’s own expertise. This raises a deeper epistemic problem: clinicians differ markedly in skill, training, experience, and judgment. An AI model that outperforms the ‘average’ doctor may not outperform this doctor.^85^ But current AI systems rarely provide comparative performance metrics, and even when such metrics are available, they seldom isolate the relevant dimensions of clinical expertise. Thus, to argue that a clinician was negligent in not deferring to an AI system requires showing not only that the AI was statistically superior in general, but that its superiority was *decisive in the context of this case, with this patient, and this doctor*. That threshold is rarely attainable in practice.

Accordingly, imposing a general duty to adopt or follow statistically superior AI risks collapsing meaningful differences in clinical competence. It substitutes aggregate performance data for the contextual specificity that negligence traditionally requires. AI holds considerable promise, yet its integration into medical practice must be assessed by standards that recognise the context of human judgment and the diversity of clinical expertise. Given the central role of reason-giving in professional accountability, the law must be wary of treating predictive accuracy as a proxy for professional responsibility. What matters, in the end, is not just whether the machine is right, but whether the *clinician had reason to trust it*—and that is a judgment which neither algorithms nor their developers can safely make on the clinician’s behalf.

## VI. Structuring a duty to use AI in clinical diagnosis

If predictive accuracy alone cannot ground a legal duty of care, the question becomes under what institutional and epistemic conditions AI use may properly form part of responsible medical practice. A clinician can only reasonably be expected to anticipate and assess the risks of a system where adequate institutional infrastructure and cognitive support exist to interpret and use its outputs safely. Responsibility for determining when AI tools enter the clinical standard of care must therefore be shared and structured. It cannot rest solely on the individual practitioner. Rather, it should be guided by institutional and statutory processes; a process-based approach to defining professional standards. Organizations such as NICE or the Royal Colleges already play this role in clinical negligence law, with their guidance forming part of the evidentiary framework for what constitutes responsible medical practice under *Bolam*.^86^ Unlike initial regulatory approval, which focuses primarily on safety and technical compliance, NICE endorsement signals that a tool has met robust evidence-based standards for clinical effectiveness and cost-efficiency,^87^ typically through evaluation mechanisms such as the Medical Technologies Evaluation Programme^88^ or the Digital Health Technologies Framework.^89^ These bodies are well-placed not only to assess the clinical efficacy of AI systems, but also to consider their reliability across contexts, their ethical ramifications, and their appropriateness in light of resource constraints. Courts may justifiably treat deviations from this form of guidance as presumptively negligent, subject to reasoned justification.

Still, even in this more institutional model, professional discretion must remain. To that end, institutional endorsement must be accompanied by a minimum standard of algorithmic *transparency*—a threshold level of disclosure that allows users to evaluate the model’s intended scope, data provenance, and performance limitations in advance of use. This kind of ex ante transparency is indispensable to both sound clinical judgment and legal defensibility. Without it, clinicians are deprived of the very information that would enable them to act reasonably in deploying, or declining to deploy, an AI tool.^90^ Technical tools such as feature importance analysis, partial dependence plots, and rule extraction methods serve to render even complex models more interpretable. Feature importance analysis^91^ helps quantify the relative influence of each feature on the model’s outputs. Partial dependence plots^92^ show how a specific feature’s values influence predictions while keeping other features constant. Rule extraction methods^93^ aim to convert complex machine learning models into a set of understandable rules. Such tools do not eliminate opacity, but they mitigate it. And in doing so, they reduce not only clinical hesitancy but also the evidentiary burdens courts must navigate in post hoc inquiries into liability.

Where a tool is institutionally recommended, and where adequate transparency has been provided, the default expectation will be that a clinician uses it. If she does not, she will be required to explain why. That explanation will be judged by reference to the circumstances^94^: Was the tool unavailable? Was the patient’s case materially outside the model’s design domain? Was there informed patient refusal? What will not suffice, however, is mere unease or anecdotal dissatisfaction. Criticism must be grounded in evidence—evidence, for instance, of systematic bias in the model’s training data or a demonstrated mismatch with the clinician’s patient population.

Economic analysis does not point inexorably in the opposite direction. Indeed, AI is widely expected to enhance clinical efficiency by accelerating diagnosis and treatment, reducing redundant testing, and minimizing human error. Over time, it may also yield cost savings by optimizing resource allocation across healthcare systems.^95^ So the question naturally arises: isn’t better average performance tantamount to better care? The answer is: not necessarily. The law’s concern is not with the average, but with the particular. While statistical performance bears on the ‘size of the risk’ in negligence calculus, it is only one input in a broader calculus^96^—alongside the potential severity of harm, the cost of taking precautions, and the utility of the conduct in question.^97^ This framework, famously expressed by Learned Hand’s formula (B < PL),^98^ has since evolved into a marginal analysis of cost-justified care.^99^ But economic models depend on reasonably knowable inputs.^100^ And here, AI’s opacity poses a serious problem. Without access to the underlying data, algorithmic architecture, or calibration thresholds that would enable a meaningful estimation of risk in the individual case, the economic model stalls. So even the economist must conclude: the law cannot mandate what cannot be meaningfully evaluated. Furthermore, purely utilitarian reliance on aggregate accuracy risks legitimizing practices where the overall population benefits from AI use, but vulnerable subgroups—such as racial minorities, patients with rare conditions, or others underrepresented in training data—bear disproportionate risks of misdiagnosis or mistreatment. This would lead to a statistical calculus in which the few are sacrificed for the many. Instead, we must reinforce a standard of care that respects the rights and dignity of each patient.

In sum, a legal duty to use AI in clinical care cannot rest on statistical superiority alone. It must instead emerge from a tripartite foundation: regulatory approval, institutional endorsement, and operational transparency. These conditions ensure that reliance on the system is capable of constituting responsible medical practice within the meaning of *Bolam*, while also satisfying *Bolitho’s* requirement that such practice withstand logical scrutiny. Only where an AI tool has been validated in this way can clinicians make informed, defensible decisions, and only then can the law coherently treat non-use as a potential deviation from the standard of care.

## VII. Structuring duties to rely on (or disregard) AI outputs in clinical decision-making

The complexity, limited interpretability, and probabilistic nature of AI tools create not only challenges for doctors deciding whether to use a device, but also uncertainty about how to engage with its output.

### A. Liability for not following AI predictions

Parallel to the requirements outlined above, I suggest that one does not arrive at liability merely because a doctor declined to follow the prediction of an AI system. Rather, liability begins to take shape only when certain normative and institutional preconditions are satisfied.

First, any claim that a doctor should have followed an AI recommendation presupposes that the device’s use was itself lawful and clinically appropriate in the circumstances. If deployment would already fall below the standard of care (because the device lacked regulatory approval, exceeded its intended scope, or was unsuitable for the patient), no duty to follow its outputs can arise.

Secondly, clinicians cannot reasonably be expected to assess the technical architecture and statistical validity of complex AI systems independently. Liability for declining to follow an AI-generated recommendation should therefore turn on whether use of the tool has been authoritatively endorsed as part of responsible medical practice. As stated above, endorsement by bodies such as NICE is relevant evidence within the *Bolam* framework: it helps establish whether a responsible body of medical opinion regards reliance on the system as proper in defined circumstances. Compliance will ordinarily support a finding of reasonableness; departure is not per se negligent, but it calls for a clinically reasoned explanation. Where a validated system operates within its intended scope and produces a high-confidence recommendation consistent with prevailing standards, a reasonable clinician may be expected at least to engage with, and where appropriate interrogate, its advice.

As adoption stabilizes, formal endorsement may give way to professional custom. If an AI model becomes generally accepted as reliable for specified applications, its integration may come to reflect ordinary competent practice. At that point, the orthodox *Bolam*–*Bolitho* analysis applies in the usual way: conformity with the system’s outputs may evidence responsible practice, while departure must be justified by reference to patient-specific considerations or recognized limitations of the tool, and the underlying professional view must remain logically defensible.^101^

Thirdly, institutional endorsement alone is insufficient. The output must also be capable of meaningful post hoc interpretation. Interpretability does not require full algorithmic transparency, but it does require that the clinician be able to understand, in clinically relevant terms, why a particular recommendation was produced. *Ex post interpretability* tools such as feature importance analysis, local surrogate models (eg LIME^102^), or SHAP value visualization^103^ can provide this bridge between statistical computation and clinical reasoning. Saliency maps, often used in medical imaging, visually highlight the regions of input data that most influenced the model’s decision, offering an intuitive mechanism for tracing algorithmic reasoning in high-stakes clinical contexts.^104^ Without some such bridge, the demand that a clinician defer to the system risks detaching legal responsibility from reasoned professional judgment. A recommendation that cannot be interrogated cannot readily ground a duty of compliance.

This interpretability requirement is not a technocratic flourish; it responds to the deeper epistemic asymmetries between humans and machines. AI models often excel at recognizing statistical patterns but lack an integrative understanding. For example, a radiologist reviewing a flagged scan must interpret not merely the flagged region, but how it fits within a wider clinical picture. Where the AI recommendation lacks such contextual coherence, or cannot be reconciled with the clinician’s judgement, it is the clinician who must decide, and who must do so in a way that can be explained. This, in turn, requires robust documentation,^105^ not just of the decision taken, but of the interpretive path by which the AI’s output was accepted, modified, or set aside.

The importance of AI interpretability is underscored by the cognitive limits of physicians under probabilistic conditions. As in the classic case of base rate neglect,^106^ even well-trained professionals may over-interpret a high-sensitivity test result for a low-prevalence condition. At the same time, interpretability tools and confidence scores, though intended to guide human judgment, do not invariably augment it. They may overwhelm rather than assist,^107^ particularly where poorly aligned with the task at hand or with human cognitive limits. Misplaced trust in probabilistic confidence, or undue scepticism, can both distort clinical decisions. Hence, human–AI collaboration must be underwritten by interfaces designed for practical intelligibility by those who must act on the system’s advice. Practical intelligibility requires interfaces that translate statistical outputs into clinically usable signals. For example, instead of merely presenting a probability score (eg ‘malignancy risk: 0.72’), an interface might display (i) the key patient-specific features driving that estimate (eg lesion size, growth rate, biomarker levels), (ii) the model’s confidence interval or uncertainty band, and (iii) whether the patient falls outside the distribution of the training data. Similarly, in imaging contexts, saliency maps could allow clinicians to toggle between the raw image, highlighted region, and comparative exemplars from the training set. The aim is not to replicate the model’s internal logic in full, but to provide information calibrated to clinical reasoning. These enable the doctor to ask: does this recommendation cohere with the patient’s presentation, and if not, why?

It should be noted that a crucial distinction must be drawn between AI deployed to enhance diagnostic accuracy and AI designed primarily to improve workflow efficiency, for the implications of reliance differ sharply between the two. AI may outperform human judgment in narrow diagnostic domains or improve workflow through rapid triage, as in COVID-19 screening. Yet where its role is chiefly to optimize efficiency rather than enhance diagnostic insight, reliance becomes riskier. Pre-sorting tools, for instance, may accelerate radiology queues but cannot absolve the clinician of responsibility for false positives or missed subtleties.

Thus, a legal duty to follow AI does not arise simply because the machine is more accurate on average. It arises, if at all, from the confluence of lawful approval, institutional endorsement, clinical applicability, and meaningful interpretability. Absent those conditions, the clinician retains discretion. And where that discretion is exercised reasonably and transparently, liability should not follow.

### B. Liability for following AI predictions

Imagine the following scenario: a clinician, aided by an AI-powered diagnostic tool intended for the early detection of neurological tumours, refrains from further investigation after the tool fails to flag a latent brain tumour. Treatment is delayed; harm becomes irreversible. The system’s prediction, while statistically supportable, turns out to be false. Does responsibility lie with the doctor?

As a matter of tort doctrine, reliance on an institutionally endorsed AI system, such as one recommended by NICE, places the clinician, *prima facie*, within the bounds of reasonable practice. However, clinicians must remain attentive to any limitations set out in professional guidelines or the device’s instruction manual, as well as to patient-specific considerations. Guidance may explicitly indicate when and how the outputs of certain devices, such as rapid diagnostic tools known for their high specificity (ability to correctly identify those without the disease), should be questioned or verified. If a device is used beyond its intended scope, or in complex cases for which it was not trained, the justification for relying on its output diminishes. Similarly, it could be negligent use where the AI was known to produce inconsistent outputs due to non-deterministic inference, or its performance on certain patient groups (eg racial minorities or rare conditions) was unvalidated.

Moreover, clinicians can still weigh the AI’s result against other clinical evidence, like patient history and lab tests. Where interpretability tools—such as saliency maps—generate results that contradict the prediction (eg by highlighting regions inconsistent with the suspected diagnosis), a failure to test the output using traditional methods may expose the physician to liability. Especially in cases of grave clinical consequence, the absence of a discernible rationale behind an AI system’s prediction may itself give rise to a duty of further inquiry. Where life hangs in the balance, opaque reasoning cannot be blindly trusted; conventional diagnostics may be needed to shore up or second-guess the model. And yet, the opposite also holds: if the AI’s conclusion, however inscrutable, finds independent confirmation in traditional methods—physical examination, imaging, or lab results—then reliance upon it does not, without more, transgress the bounds of reasonable care.

That the precise mechanism or likelihood of harm was uncertain does not, in itself, immunize a defendant from liability. Courts have long recognized that foreseeability operates at the level of risk category, not precise unfolding. It is not the detail of the error but the kind of error—say, diagnostic misclassification or technical failure—that renders harm foreseeable and thus potentially actionable.^108^ Yet, ‘it is not enough that there is a remote possibility that injury may occur: the question is, would a reasonable man anticipate it’.^109^ If a beneficial practice needs to be sacrificed to eliminate a minimal risk, imposing liability could be disproportionate.^110^ Where the overall benefit of an AI system was substantial, and reasonable safeguards were in place, a court may find that the risk was not sufficient to require discontinuing the system entirely; the equivalent of not having to ‘stop cricket’ in *Bolton*. That assessment concerns the tolerability of a foreseeable risk. A distinct question arises where the particular kind of misdiagnosis was not, at the relevant time, part of the system’s knowable risk profile. In early-stage deployment, or where a risk materializes only at scale without prior signal, a defendant may argue that the harm was not reasonably foreseeable as a category of error. The burden lies with claimants to establish that the harm was not some exotic outlier, but the realization of a known or knowable risk profile. They may do so through empirical counterfactuals or population-wide data showing repeated failures of the same kind. These forms of evidence reframe the incident not as freakish but as symptomatic, inviting the inference that due diligence could have prevented it.

### C. Record-keeping and adverse inference

Certain AI-related scenarios may give rise to a more readily established inference of breach and causation, particularly where a duty to maintain records exists, and no records are retained. Record-keeping obligations, deeply rooted in common law, professional ethics (eg GMC guidance), and regulatory mandates, serve a range of overlapping purposes: ensuring continuity of care, facilitating informed decision-making, providing evidence in litigation, and meeting statutory and professional requirements.

In the context of AI-assisted care, these duties acquire a statutory dimension. Article 15(1)(h) GDPR entitles individuals to meaningful information about the logic and significance of automated decisions affecting them. This effectively presupposes the availability of interpretable records, especially where these influence clinical outcomes or triage decisions. For high-risk systems, Article 26(5) and (6) of the AI Act oblige deployers to monitor the operation and keep the logs automatically generated by that system for at least 6 months. In the EU, failure to meet these obligations may expose clinicians or institutions not only to regulatory sanctions but also to tortious liability.^111^

The asymmetry of informational control is not a procedural inconvenience but a substantive feature of many modern wrongs. Courts may, and often do, take the absence of a plausible explanation or documentary transparency as a signal—especially where the failure to explain lies with the party best positioned to do so. Where evidence is missing, claimants frequently invoke the doctrine of *res ipsa loquitur*.^112^ If a foreseeable harm occurs within a system under the defendant’s control, and the precise mechanism lies within the defendant’s knowledge, the court may infer breach unless the defendant shows that reasonable precautions were taken.^113^

In the AI context, however, these requirements will often be difficult to satisfy. Algorithmic error does not, without more, ‘speak for itself’, since false positives and negatives occur even in validated systems. Control is typically diffuse across developers, model customizers, institutions, and clinicians. And where the system’s internal processes are opaque, claimants may struggle to identify the kind of ‘incontrovertible facts’ traditionally required to ground the inference.

That said, courts have long recognized that where one party’s failure to create or preserve records generates evidential uncertainty, that uncertainty may weigh against them. The case law in *Keefe v The Isle of Man Steam Packet Co*^114^ and *Shawe-Lincoln v Neelakandan*^115^ illustrates how courts may draw adverse inferences from evidentiary gaps, even absent a formal reversal of the burden of proof.^116^ In *Keefe*, the employer’s failure to conduct mandatory noise assessments led the court to assess the claimant’s evidence more favourably and to view the defendant’s submissions with scepticism.^117^ This aligns with the principle in *Armory v Delamirie*,^118^ where a party’s failure to preserve key evidence justified judicial inferences adverse to their interests. In *Shawe-Lincoln*, the court emphasized that whether an adverse inference may be drawn from missing evidence depends on the surrounding context, including the proximity between the alleged breach and the missing material, and whether a duty to preserve that material existed. In the absence of such a duty, the mere non-production of a call log did not justify drawing the inference contended for by the claimant.

The question, then, is whether these evidential principles may extend to AI-enabled systems. Their applicability may depend, first, on whether a breach of duty underpins the absence of evidence (eg a failure to retain model outputs or decision paths); and secondly, on whether the missing information falls within the protective scope of that duty. The applicability of adverse inference principles does not depend exclusively on statutory logging obligations, but where regulatory regimes expressly mandate monitoring or documentation of AI outputs, the case for inference-drawing is significantly strengthened. Like unrecorded clinical observations in *Shawe-Lincoln*, opaque AI systems risk creating evidentiary voids. The principle that those best placed to preserve evidence must do so extends to AI integration. If the use of AI contributes to the claimant’s evidential deficit—whether because the system operates as a technical black box whose internal pathways are neither visible nor preserved unless deliberately logged, or because intermediate outputs are deleted and input data inadequately documented—courts may adjust their evidentiary expectations accordingly. Where clinicians deploy AI systems, they must ensure procedural mechanisms exist to record not only the outputs but also the interpretive steps taken in response. Without interpretability tools or records of how AI advice was weighed against clinical facts, courts are left guessing—and under *Shawe-Lincoln*, that may work against the defendant. While this does not necessarily entail a formal reversal of the burden of proof, it could lead to a judicial *recalibration* of inference-drawing: the party who uses the black box may not be permitted to hide inside it.

It should be noted that, under EU tort principles, including the principle of effectiveness, national courts may be required to interpret procedural rules in a manner that does not render the claimant’s rights under EU law practically impossible to enforce. The CJEU’s decision in *Sanofi Pasteur^119^* illustrates that evidentiary presumptions can be necessary to preserve claimants’ rights, particularly where direct proof is unattainable. Where transparency, record-keeping, and documentation requirements for high-risk AI systems under the AI Act are not complied with, *Sanofi* provides support for the argument that claimants should not be penalized for the resulting evidentiary opacity. Such reasoning—central to the now-withdrawn AILD^120^—provides support for allowing circumstantial evidence, probabilistic reasoning, *res ipsa loquitur*, and rebuttable presumptions in complex, technologically mediated claims where traditional forms of direct evidence are unavailable. The opacity or non-explainability of AI systems must not render claimants’ rights practically ineffective.

## VIII. Resolving disagreement between technology and the physician

Indeed, endorsement does not eliminate the possibility of conflict between professional judgment and algorithmic output. And when such conflict arises, it might not be resolvable by pointing to metadata or leaning on post hoc interpretability tools. What is needed is not just more information but a structured procedural framework—a way of navigating disagreement between human and machine that preserves the normative integrity of clinical care.

Several models suggest themselves.^121^ Algorithmic adjudication offers consistency and speed by analysing inputs through standardized models, yet it risks marginalizing contextual sensitivity. Conversely, a second-opinion physician review introduces human expertise and context sensitivity, but is resource-intensive and may itself introduce subjectivity. Shared decision-making with the patient promotes transparency and autonomy but may prove problematic where patients lack sufficient understanding of either the clinical context or the AI system. Patients cannot reasonably be asked to arbitrate between rival epistemic authorities when they are not equipped to assess either. The clinician, as fiduciary, remains the party charged with ensuring that decisions reflect the patient’s best interests, not simply the model’s statistical forecasts.

The choice of resolution model will depend on the clinical context. In high-stakes scenarios, such as oncology or emergency care, conflict protocols could involve automatic escalation to human review, particularly where the AI system indicates low confidence or high risk. A hybrid model, combining algorithmic analysis with a second medical opinion, may offer a pragmatic balance between objectivity and clinical judgment. Alternatively, differing recommendations could be disclosed to the patient and used to support a collective decision between physician and patient—provided, of course, that such dialogue is well-supported and well-informed.

NICE already supplies mechanisms for handling intraprofessional disputes over what constitutes a patient’s best interests. These include advocacy, peer review, and ethics consultation.^122^ There is no principled reason why such mechanisms could not be extended (mutatis mutandis) to disputes between clinicians and algorithms. Indeed, as the role of AI in clinical care expands, institutions may need to develop standing panels, drawing from medicine, data science, law, and ethics, to ensure that AI deployment remains anchored in judgment, not merely computation.

## IX. Conclusion

The integration of AI into clinical practice promises gains in diagnostic accuracy, efficiency, and systemic coordination. Yet negligence law cannot equate statistical superiority with legal obligation. Regulatory certification, whether CE or UKCA marking, permits clinical deployment; it does not, without more, mandate reliance. At present, doctors are not under a general duty to follow AI recommendations. A duty may arise only where the tool is lawfully deployed, institutionally endorsed (such as through NICE guidance) and capable of meaningful interpretation within the clinical context. Where those conditions are satisfied, deviation may require justification. Conversely, blind reliance on an output that contradicts clinical indicators or lacks intelligible rationale may itself fall below the standard of care.

The framework advanced in this article preserves clinical judgment while recognizing the epistemic constraints under which clinicians operate. It reallocates part of the justificatory burden to institutions best placed to monitor and explain AI systems, rather than isolating responsibility at the bedside. In doing so, it equips courts with principled criteria through which to assess breach and causation without distorting established doctrine. Above all, it maintains the central insight of negligence law: responsibility must remain tethered to reasoned human judgment.

Residual risks will persist. AI systems are probabilistic, adaptive, and in some instances non-deterministic. Harm may occur in ways that resist clean attribution to any single actor. The complexity in allocating responsibility is particularly acute with general-purpose AI systems that are developed, fine-tuned,^123^ integrated, and deployed by multiple actors. In such distributed systems, harm may emerge from a combination of design choices, training data, local adaptation, procurement decisions, and clinical use. This calls for careful attention to which actor exercised material control over the relevant risk, and who was best placed to monitor and mitigate it. However, whether difficulties of attribution amount to a true ‘liability gap’ depends on normative judgments about when damage recovery ought to be available.

Where evidential opacity renders attribution systematically impracticable, more structural responses may warrant consideration. Proportionate liability regimes or targeted no-fault compensation schemes could, in defined domains, offer a more coherent policy response than pressing fault-based doctrine into roles it was not designed to perform.^124^ Exceptional causation doctrines (such as material contribution to harm, and in tightly confined circumstances material increase in risk) retain their place, but they are not general solutions to technological complexity.^125^ Any broad recalibration of causal or apportionment principles would be a policy decision and likely require legislative intervention rather than incremental judicial development. It should be noted, however, that this article is concerned primarily with the structuring of duty and breach in AI-mediated clinical decision-making, rather than offering a full account of causation or proportionate liability, which raises distinct systemic questions.

As AI systems become more reliable and more deeply embedded in clinical practice, professional expectations will inevitably evolve. What is presently optional may, in time, form part of ordinary competence. Yet such evolution must remain sensitive to context and to disparities in access and institutional support. The law must preserve a framework in which innovation enhances, rather than erodes, accountability and trust. However sophisticated the system, the demand that a clinician stand ready to justify a decision in reasons intelligible to others remains undiminished. AI may alter the sources of judgment, but it does not relieve the doctor of the burden of giving one.

## Footnotes

1 For example, the Microsoft AI Diagnostic Orchestrator (MAI-DxO) demonstrates a diagnostic accuracy of up to 85 per cent on case studies where experienced human physicians achieve accuracy rates less than one-quarter as high. Additionally, MAI-DxO reaches correct diagnoses at a lower cost: Microsoft, ‘The Path to Medical Superintelligence’ (*Microsoft AI*, 2025) <https://microsoft.ai/new/the-path-to-medical-superintelligence/> accessed 15 October 2025.

2 Google Health’s AI already outperformed human experts in mammogram interpretation in 2020, significantly reducing false negatives and positives: Scott M McKinney and others, ‘International Evaluation of an AI System for Breast Cancer Screening’ (2020) 577 Nature 89 <https://doi.org/10.1038/s41586-019-1799-6> accessed 2 July 2025. Transformer-based neural network models outperformed both expert and non-expert examiners in ultrasound detection of ovarian cancer: Frederik Christiansen and others, ‘International Multicenter Validation of AI-Driven Ultrasound Detection of Ovarian Cancer’ (2025) 31 Nature Medicine 189 <https://doi.org/10.1038/s41591-024-03329-4> accessed 15 October 2025.

3 Department of Health and Social Care, ‘Fit for the future: 10 Year Health Plan for England–executive summary’ (*gov.uk*, updated 30 July 2025) <https://www.gov.uk/government/publications/10-year-health-plan-for-england-fit-for-the-future/fit-for-the-future-10-year-health-plan-for-england-executive-summary> accessed 17 February 2026.

4 For example, leaving unresolved the extent of physicians’ deference to AI, the means of interrogating AI, and the role of regulatory standards as this ‘will unfold in courts and other arenas’: W Nicholson Price II and others, ‘Liability for Use of Artificial Intelligence in Medicine’ in Barry Solaiman and I Glenn Cohen (eds), *Research Handbook on Health AI and the Law* (Edward Elgar Publishing 2024) 155. Others have said ‘once a machine is demonstrably superior to human diagnosticians, malpractice law will require the use of the superior technology in certain sectors of medical diagnostics’: A Michael Froomkin and others, ‘When AI Outperform Doctors’ (2019) 61 Arizona Law Review 33, 36.

5 ‘Replace[ment]’ explicitly assumed by M Geistfeld and others, ‘Comparative Law Study on Civil Liability for Artificial Intelligence’, in M Geistfeld and others (eds), *Civil Liability for Artificial Intelligence and Software* (De Gruyter 2023) 11; similar consideration in E Marchisio, ‘In support of ‘no-fault’ civil liability rules for artificial intelligence’ (2021) 1 SN Social Sciences 54; explicitly excluding the user and only looking at the operator: C Wendehorst, ‘Product Liability or Operator Liability for AI—What is the Best Way Forward?’ in Lohsse and others (eds), *Liability for AI: Münster Colloquia on EU Law and the Digital Economy VII* (Hart Publishing 2023) 114–115.

6 See chs II–VIII of the Artificial Intelligence Act (European Parliament, 13 March 2024), <www.europarl.europa.eu/doceo/document/TA-9-2024-0138_EN.pdf> accessed 15 October 2025.

7 See K Sokol and others, ‘Artificial intelligence should genuinely support clinical reasoning and decision making to bridge the translational gap’ (2025) 8 NPJ Digital Medicine 345 <https://doi.org/10.1038/s41746-025-01725-9> accessed 15 October 2025.

8 Recital 15 of the Proposal for a Directive of the European Parliament and of the Council on Adapting Non-Contractual Civil Liability Rules to Artificial Intelligence (AI Liability Directive) (*EUR-Lex*, 28 September 2022) <https://eur-lex.europa.eu/legal-content/EN/TXT/PDF/?uri=CELEX:52022PC0496> accessed 15 October 2025.

9 For a rebuttal to the statement in Recital 15 of the AILD, see P Hacker, ‘The European AI Liability Directives–Critique of a Half-Hearted Approach and Lessons for the Future’ (2023) 51 Computer Law & Security Review 105871, 19.

10 Using the term ‘foreseeability’: Selbst, ‘Negligence and AI’s Human Users’, 100 BUL Review (2020) 1342; see also Amelie Berz, ‘From Optional to Obligatory: Why AI’s Statistical Superiority Doesn’t Dictate Tort Law Duties’ (*Oxford Business Law Blog*, 23 July 2024), <https://blogs.law.ox.ac.uk/oblb/blog-post/2024/07/optional-obligatory-why-ais-statistical-superiority-doesnt-dictate-tort-law> accessed 15 October 2025.

11 See Phillip Morgan, ‘Tort Law and AI—Vicarious Liability’ in E Lim and P Morgan (eds), *The Cambridge Handbook of Private Law and Artificial Intelligence* (CUP 2024) 138ff.

12 See Barry Solaiman and Abeer Malik, ‘Regulating Algorithmic Care in the European Union: Evolving Doctor–Patient Models through the Artificial Intelligence Act and the Liability Directives’ (2025) 33 Medical Law Review fwae033.

13 It should be noted that the term ‘standard of care’ appears to be used both to describe the benchmark a defendant is measured against (‘reasonable person standard’) and the actions required as part of the duty of care (eg a reasonable person would ‘use AI’ or ‘not use AI’). The former stays the same; the latter will change as society’s legitimate expectations of what is ‘reasonable’ changes.

14 *Blyth v Birmingham Waterworks Company* [1856] 11 Ex Ch 781 (‘reasonable man, guided upon those considerations which ordinarily regulate the conduct of human affairs’; ‘prudent and reasonable man’).

15 S Grundmann, *Münchener Kommentar zum Bürgerlichen Gesetzbuch* (9th edn, CH Beck 2022), BGB s 276 para 55; G Wagner, *Münchener Kommentar zum Bürgerlichen Gesetzbuch* (8th edn, CH Beck 2020), BGB s 823 para 42.

16 R Posner, *Economic analysis of law* (9th edn, Wolters Kluwer Law & Business 2014) 196, who writes that the approach ‘is justified *only* by the costs of individualized measurement’ (emphasis by the author).

17 *Wells v Cooper* (1958) 2 All ER 527.

18 *Nader v Urban Transit Authority of NSW* (1985) 2 NSWLR 501.

19 *Baker v Quantum Clothing Group* [2011] UKSC 17.

20 *Bolam v Friern Hospital Management Committee* [1957] 1 WLR 582, 587 (‘*Bolam*’).

21 ibid.

22 [1985] 1 All ER 635.

23 [1998] AC 232 (‘*Bolitho*’).

24 *Bolitho* 241–42 (Lord Browne-Wilkinson): ‘The use of these adjectives—responsible, reasonable and respectable—all show that the court has to be satisfied that the exponents of the body of opinion relied upon can demonstrate that such opinion has a logical basis’.

25 Gideon Parchomowsky and Alex Stein, ‘Torts and Innovation’ (2008) Michigan Law Review 285, 288 <https://repository.law.umich.edu/cgi/viewcontent.cgi?article=1365&context=mlr> accessed 14 July 2023.

26 *Marriott v West Midlands HA* [1999] Lloyd’s Rep Med 23.

27 Goudkamp and Nolan, *Winfield & Jolowicz on Tort* (20th edn, Thomson Reuters 2020), para 6.019–6.023.

28 *Bolitho* 243 (Lord Browne-Wilkinson, emphasis added).

29 *Roe v Minister of Health* [1954] 2 QB 66.

30 C*hester v Afshar* [2004] UKHL 41; *Montgomery v Lanarkshire Health Board* [2015] UKSC 11.

31 Heywood and others, ‘Informed Consent in Hospital Practice’ (2010) Medical Law Review 152.

32 NHS, ‘Overview: Consent to Treatment’ (*NHS*, 8 December 2022) <www.nhs.uk/conditions/consent-to-treatment/> accessed 15 October 2025.

33 See General Medical Council, ‘Decision making and consent’ 5 <www.gmc-uk.org/-/media/documents/gmc-guidance-for-doctors—decision-making-and-consent-english_pdf-84191055.pdf> accessed 15 October 2025.

34 *Montgomery v Lanarkshire Health Board* [2015] UKSC 11 which overturned *Sidaway v Board of Governors of the Bethlem Royal Hospital and the Maudsley Hospital* [1985] AC 871.

35 General Medical Council (n 33) 16.

36 *Montgomery v Lanarkshire Health Board* [2015] UKSC 11; even unlikely yet severe risks need to be conveyed, eg a 1–2 per cent risk of paralysis in a surgical procedure: *Chester v Afshar* [2004] UKHL 41.

37 *Miller v Ministry of Pensions* [1947] 2 All ER 372, 374 (Denning J): ‘the evidence is such that a tribunal can say ‘we think it more probable than not’; *F H v McDougall* [2008] 3 SCR 41, 43: the BPR requires a ‘51% probability’; Sandy Steel, ‘Justifying Exceptions to Proof of Causation in Tort Law’ (2015) 78 The Modern Law Review 729, 758.

38 *Wilsher v Essex Area Health Authority* [1988] 1 AC 1074, where the House of Lords overturned the respective first instance decision. They had misinterpreted *McGhee v National Coal Board* [1972] UKHL 7, 1 WLR 1.

39 *C v North Cumbria University Hospitals NHS Trust* [2014] EWHC 61 (QB) at [84].

40 *David Price v Cwm Taf University Health Board* [2019] EWHC 938 (QB) at [22].

41 B Jack Copeland, ‘Artificial Intelligence’ (*Encyclopedia Britannica*, 5 June 2023) <www.britannica.com/technology/artificial-intelligence> accessed 15 October 2025.

42 William Hosch, ‘Machine Learning’ (*Encyclopedia Britannica*, May 25 2023) <www.britannica.com/technology/machine-learning> accessed 15 October 2025.

43 AI for Everyone, ‘What are Rule-Based Systems in AI?’ (*Autoblocks*) <www.aiforanyone.org/glossary/rule-based-system> accessed 15 October 2025.

44 R A Miller and others, ‘Ethical and Legal Issues related to the Use of Computer Programs in Clinical Medicine’ (1985) 102 Annals of Internal Medicine 529, 535.

45 Department of Health and Social Care, ‘AI to be Trialled across NHS Screening to Speed Up Disease Diagnosis’ (*NIHR*, 26 September 2025) <https://www.nihr.ac.uk/news/ai-be-trialled-across-nhs-screening-speed-disease-diagnosis>; NHS England, ‘NHS Artificial Intelligence (AI) Trial to Diagnose Prostate Cancer up to a Month Laster’ (28 October 2025) <https://www.england.nhs.uk/2025/10/nhs-artificial-intelligence-ai-trial-to-diagnose-prostate-cancer-up-to-a-month-faster/>; NHS England, ‘NHS Launches Trailblazing AI and Robot Pilot to Spot Lung Cancer Sooner’ (27 January 2026) <https://www.england.nhs.uk/2026/01/nhs-launches-trailblazing-ai-and-robot-pilot-to-spot-lung-cancer-sooner/>; NHS England, ‘Life-Changing AI Support Helping Stroke Patients Get a Second Chance’ (2 December 2025) <https://www.england.nhs.uk/2025/12/life-changing-ai-support-helping-stroke-patients-get-a-second-chance/> all accessed 17 February 2026.

46 Nikolas Zöller and others, ‘Human–AI Collectives Most Accurately Diagnose Clinical Vignettes’ (2025) 122 Proceedings of the National Academy of Sciences of the United States of America e2426153122.

47 David Talby, ‘The Accuracy Limits of Data-Driven Healthcare’ (*Forbes*, 16 February 2022) <www.forbes.com/sites/forbestechcouncil/2022/02/16/the-accuracy-limits-of-data-driven-healthcare/?sh=1434aef54623> accessed 15 October 2025.

48 Matthew Fenech and Olly Buston, ‘AI in Cardiac Imaging: A UK-Based Perspective on Addressing the Ethical, Social, and Political Challenges’ (2020) 7 Frontiers in Cardiovascular Medicine 4.

49 Boris Babic and others, ‘Beware Explanations from AI in Health Care’ (2021) 373 Science 6552, 284.

50 Kerr I and others, ‘Robots and Artificial Intelligence in Health Care’ in Erdman J and others (eds), *Canadian Health Law and Policy* (5th edn, LexisNexis Canada, 2017) 266.

51 For further explanations on these methods, see IBM, ‘What is Explainable AI?’ (*Think*, 29 March 2023) <https://www.ibm.com/think/topics/explainable-ai> accessed 4 September 2025.

52 Alejandro Barredo Arrieta and others, ‘Explainable Artificial Intelligence (XAI): Concepts, Taxonomies, Opportunities and Challenges toward Responsible AI’ (2020) 58 Information Fusion 82, 90 <https://arxiv.org/abs/1910.10045> accessed 15 October 2025.

53 s 13 of the UK MDR 2002.

54 Data Protection Act 2018 <www.legislation.gov.uk/ukpga/2018/12/contents/enacted> accessed 15 October 2025.

55 EU GDPR <https://gdpr.eu/tag/gdpr/> accessed 15 October 2025.

56 Art 20(1) of the EU MDR.

57 The AI Act entered into force on 1 August 2024; most of its obligations begin to apply from 2 August 2026. For more information, see ‘The AI Act Explorer’ <https://artificialintelligenceact.eu >

58 See Philipp Hacker, Amelie Berz, and Johann Cordes, ‘Transparency Requirements in the Use of Generative AI’ (2024) 23 GRUR 1777.

59 Under s 10 of the UK MDR 2002 and art 10(6), 20 of the EU MDR, AI-enabled tools must undergo a conformity assessment that evaluates design, risk analysis, and performance before acquiring CE marking.

60 AI & Digital Regulations Service for Health and Social Care, ‘Thinking about whether a medical device will meet your needs’ (NHS, 7 June 2023) <https://www.digitalregulations.innovation.nhs.uk/regulations-and-guidance-for-adopters/all-adopters-guidance/thinking-about-whether-a-medical-device-will-meet-your-needs/> accessed 15 October 2025.

61 While breach of statutory duty does not of itself give rise to a private law cause of action (*Stovin v Wise* [1996] AC 923, 936–37), the existence and scope of any common law duty of care may be influenced by the statutory framework within which the defendant acts (*X (Minors) v Bedfordshire CC* [1995] 2 AC 633, 739). The UK MDR 2002 and EU MDR establish minimum safety and conformity standards designed to protect patients from defective or insufficiently validated devices. A clinician who deploys, or an institution that procures, a device in knowing disregard of such requirements will therefore face considerable difficulty in demonstrating that their conduct satisfied the Bolam–Bolitho standard.

62 Froomkin and others (n 4) 1448; about patient’s autonomy and doctors’ decisions, I Glenn Cohen, ‘Informed Consent and Medical Artificial Intelligence’ (2020) 108 Georgetown Law Journal 1425, 1457.

63 Cohen (n 62) 1443–1447.

64 General Medical Council (n 33) 9.

65 See Goudkamp and Nolan (n 27), para 5-033. An exception might be the hypothetical case where a doctor denies treatment before a duty of care arises, which cannot be considered in the limited scope of this work.

66 AI diagnostics would follow the path of many other digital technologies if they exhibit high fixed costs, referring to the costs incurred regardless of the number of service or output units, but relatively low marginal costs: Froomkin and others (n 4) 64.

67 AI & Digital Regulations Service for Health and Social Care, ‘All Adopters’ Guidance: Regulations and Guidance for Adopters of Digital Technologies in Health and Social Care’ (NHS) <https://www.digitalregulations.innovation.nhs.uk/regulations-and-guidance-for-adopters/all-adopters-guidance/> accessed 15 October 2025.

68 Tanmoy Sarkar Pias and others, ‘Low responsiveness of Machine Learning Models to Critical or Deteriorating Health Conditions’ (2025) 5 Communications Medicine 62.

69 ibid.

70 Marina Chugunova and Daniela Sele, ‘We and It: An Interdisciplinary Review of the Experimental Evidence on How Humans Interact with Machines’ (2022) 99 Journal of Behavioral and Experimental Economics 101897.

71 Romain Cadario and others, ‘Understanding, Explaining, and Utilizing Medical Artificial Intelligence’ (2021) Nature Human Behaviour 1636.

72 Chugunova and Sele (n 70).

73 Cohen (n 62) 1443.

74 Hadrien Pouget and Ranj Zuhdi, ‘AI and Product Safety Standards Under the EU AI Act’ (*Carnegie Endowment*, 5 March 2024), <https://carnegieendowment.org/research/2024/03/ai-and-product-safety-standards-under-the-eu-ai-act?lang=en> accessed 15 October 2025.

75 Tom Brown and others, ‘Language Models are Few-Shot Learners’ (2020) 33 Advances in Neural Information Processing Systems 1877.

76 Ronald Allen and Alex Stein, ‘Evidence, Probability, and the Burden of Proof’ (2013) 55 Arizona Law Review 557, 567.

77 [2002] EWHC 1420.

78 *Sienkiewicz v Greif Ltd* [2011] UKSC 10.

79 L Jonathan Cohen, *The Probable and the Provable* (OUP 1977), § 30; David Wasserman, ‘The Morality of Statistical Proof and the Risk of Mistaken Liability’ (1991) 13 Cardozo Law Review 935, 942–943.

80 Nate Adams, ‘Bare Statistical Evidence and the Right to Security’ (2023) 24 Journal of Ethics and Social Philosophy 291–311.

81 Lord Phillips in *Sienkiewicz* at [94].

82 Austin Bradford Hill, ‘The Environment and Disease: Association or Causation?’ (1965) 58 Proceedings of the Royal Society of Medicine 295–300.

83 *Ministry of Defence v Wood* [2011] EWCA Civ 792, [61]; *XYZ v Schering Health Care Ltd* [2002] EWHC 1420, [302]; *Reay v British Nuclear Fuels Plc* (1994) 5 Med L R 01.

84 About the general difficulties in comparison: JE Hans Korteling and others, ‘Human versus Artificial Intelligence’ (2021) Frontiers in Artificial Intelligence 4, 1–11.

85 For a respective empirical study, see Harvard Medical School, *Does AI Help or Hurt Human Radiologists? Performance Depends on the Doctor* (16 November 2023) <https://hms.harvard.edu/news/does-ai-help-or-hurt-human-radiologists-performance-depends-doctor> accessed 15 October 2025.

86 For examples of such guidance, see National Institute for Health and Care Excellence (NICE), *Guidance on Artificial Intelligence <*https://www.nice.org.uk/guidance/published?q=artificial+intelligence> accessed 15 October 2025.

87 As an example, see: ‘Artificial Intelligence (AI)-Derived Software to Help Clinical Decision Making in Stroke’ (*NICE*, 23 January 2024, updated 2 May 2024), <https://www.nice.org.uk/guidance/dg57/chapter/1-Recommendations> accessed 15 October 2025.

88 ‘Medical Technologies Evaluation Programme: Get a Medical Technology Evaluated’ (*NICE*) <https://www.nice.org.uk/about/what-we-do/our-programmes/nice-guidance/nice-medical-technologies-evaluation-programme> accessed 15 October 2025.

89 National Institute for Health and Care Excellence (NICE), *Evidence Standards Framework for Digital Health Technologies* (NICE, updated 2024) <https://www.nice.org.uk/about/what-we-do/our-programmes/evidence-standards-framework-for-digital-health-technologies> accessed 15 October 2025.

90 Respectively, according to Article 13(3)(b) of the AI Act, instructions for use of high-risk systems must specify the expected levels of accuracy, robustness, and cybersecurity of a high-risk AI system—including how these were tested and validated, as well as any conditions that may affect them.

91 Terence Shin, ‘Understanding Feature Importance in Machine Learning’ (*Towards Data Science*, 7 November 2024) <https://builtin.com/data-science/feature-importance> accessed 15 October 2025.

92 Mehul Gupta, ‘Understanding Partial Dependence Plots (PDPs)’ (*Medium*, 17 October 2022) <https://medium.com/data-science-in-your-pocket/understanding-partial-dependence-plots-pdps-415346b7e7f1> accessed 15 October 2025.

93 Tameru Hailesilassie, ‘Rule Extraction Algorithm for Deep Neural Networks: A Review’ (2016) *International Journal of Computer Science and Information Security* 14, 376 <https://arxiv.org/pdf/1610.05267.pdf> accessed 15 October 2025.

94 *Price v Cwm Taf University Health Board* [2019] EWHC 938 (QB).

95 Rabie Adel El Arab and Omayma Abdulaziz Al Moosa, ‘Systematic review of cost effectiveness and budget impact of artificial intelligence in healthcare’ (2025) 8 npj Digital Medicine 548.

96 Donal Nolan and Ken Oliphant, *Lunney & Oliphant’s Tort Law* (7th edn, OUP 2023) 171.

97 ibid 182–194.

98 *United States v Carroll Towing Co* 159 F.2d 169 (2d Cir. 1947). Learned Hand’s formulation (B < PL) is not part of English negligence doctrine and is best treated as an analytic heuristic rather than a legal test. It simply makes explicit the risk-balancing exercise that English courts already perform, albeit without mathematical formulation.

99 See Jules Coleman, ‘Book Review: The Structure of Tort Law’ (1987) 97 Yale Law Review 1233, 1234; Posner (n 16) 192 with the ‘marginal Hand Formula’ also derived by differential calculus.

100 In some contexts, a third party may be best placed to minimize systemic risk: Coleman (n 99) 1241 ff.

101 See Yew, ‘Medical AI, Standard of Care in Negligence and Tort Law’, in Ywe G and Yip M (eds), AI, Data and Private Law: Translating Theory into Practice (Hart Publishing 2021) 186. Nonetheless, we are far from that point in most domains.

102 Local Interpretable Model-Agnostic Explanations generate simplified surrogate models to approximate complex algorithms at the level of individual predictions: Giorgio Visani, ‘LIME: explain Machine Learning predictions’ (*Towards Data Science*, 18 December 2020) <https://towardsdatascience.com/lime-explain-machine-learning-predictions-af8f18189bfe> accessed 15 October 2025.

103 SHAP values, grounded in cooperative game theory, assign weighted contributions to input features: Fernando Lopez, ‘SHAP: Shapley Additive Explanations’ (*Towards Data Science*, 11 July 2021) <https://towardsdatascience.com/shap-shapley-additive-explanations-5a2a271ed9c3> accessed 15 October 2025.

104 Michel Kana, ‘Practical Guide for Visualizing CNNs Using Saliency Maps’ (*Towards Data Science*, 31 May 2021) <https://towardsdatascience.com/practical-guide-for-visualizing-cnns-using-saliency-maps-4d1c2e13aeca> accessed 15 October 2025.

105 See also Megan Prictor and others, ‘Clinical Decision Support Systems and Medico-Legal Liability in Recall and Treatment: A Fresh Examination’ (2020) 28 Journal of Law and Medicine 132, 144.

106 When a physician encounters a positive test for a rare condition such as breast cancer, the intuitive response may be to equate the sensitivity of the test with the likelihood of disease, neglecting the low base rate of the condition itself. Consider a routine mammogram administered to a woman with no symptoms and no family history of breast cancer—representing a population in which the disease has a base rate of roughly 1 per cent. The radiologist, upon receiving a positive result from a test with 90 per cent sensitivity and a 9 per cent false-positive rate, informs the patient that she likely has cancer, perhaps estimating the chance at 90 per cent or higher. In doing so, the physician commits a common but critical error: neglecting the base rate of the disease. In fact, the true probability that the patient has cancer, given the positive result, is closer to 9 per cent. See Elina Stengård and others, ‘On the Generality and Cognitive Basis of Base-Rate Neglect’ (2022) 226 Cognition 105160 <https://doi.org/10.1016/j.cognition.2022.105160> accessed 15 October 2025.

107 Vaccaro and others, ‘When Combinations of Humans and AI are Useful: A Systematic Review and Meta-Analysis’ (2024) 8 Nature Human Behaviour 229.

108 A claimant may recover if the kind of damage was foreseeable, even if the precise mechanism was not: *Hughes v Lord Advocate* [1963] AC 837. However, this is arguably a remoteness case: the ‘breach’ of duty by not anticipating the danger posed by meddling children had already been established.

109 *Bolton v Stone* [1951] AC 850, 860 (Lord Porter).

110 *ibid* 862 (Lord Normand), 866-67 (Lord Reid).

111 In Member States, safety-management duties can translate into tort exposure through domestic doctrines that incorporate regulatory standards into wrongfulness. In Germany, for example, § 823(2) BGB permits damages for violation of a protective statute (‘Schutzgesetz’); where an AI Act deployer obligation is patient-protective, material non-compliance may satisfy the unlawfulness element, subject to causation and scope-of-protection limits.

112 An early explanation can be found in *Scott v London & St Katherine Docks Company* (1865) 3 H & C 596, 601; 159 ER 665, 667 (Exch): ‘…where the thing is shown to be under the management of the defendant or his servants, and the accident is such as in the ordinary course of things does not happen if those who have the management use proper care, it affords reasonable evidence, in the absence of explanation by the defendants, that the accident arose from want of care.’ (Erle J).

113 *Ward v Tesco Stores* [1976] 1 WLR 810, 814 (LJ Lawton,), 815–16 (LJ Megaw).

114 [2010] EWCA Civ 683; argument mentioned in Steel, ‘Legal Causation and AI’, in Lim & Morgan (eds), *The Cambridge Handbook of Private Law and Artificial Intelligence* (CUP March 2024) 204–205.

115 [2012] EWHC 1150.

116 No reversal of the burden of proof: *Shawe-Lincoln v Neelakandan* at [80] and [81] (Justice Lloyd Jones).

117 *Keefe v The Isle of Man* at [19].

118 (1722) 1 Strange 505, 93 ER 664.

119 Case C-621/15 *N W and Others v Sanofi Pasteur MSD SNC and Others* ECLI:EU:C:2017:484, Judgment of the Court (Second Chamber) of 21 June 2017, at [30] – [31].

120 Article 3(5), 4(1)(a).

121 See John Banja and others, ‘When Artificial Intelligence Models Surpass Physician Performance: Medical Malpractice Liability in an Era of Advanced Artificial Intelligence’ (2022) 19 Journal of the American College of Radiology 816, 818.

122 General Medical Council (n 33) 37.

123 Fine-tuners may only adjust small slices of data, but their interventions ripple across billions of model parameters, producing unpredictable behavioural shifts that challenge legal assumptions of modular responsibility: Ananya Kumar and others, ‘Fine-Tuning Can Distort Pretrained Features and Underperform Out-of-Distribution’ (*arXiv preprint*, 21 February 2022) <https://doi.org/10.48550/arXiv.2202.10054> accessed 15 October 2025.

124 For example, Hacker and Holweg defend a layered causal-proportional allocation model across the AI value chain, under which responsibility tracks technical influence, control, and risk contribution: Philipp Hacker and Matthias Holweg, ‘The Regulation of Fine-Tuning: Federated Compliance for Modified General-Purpose AI Models’ (2026) 60 Computer Law & Security Review 106234. Morgan (n 11) proposes statutory vicarious liability under English law.

125 The Supreme Court reaffirmed that the *Fairchild* ‘material increase in risk’ principle constitutes a narrowly confined exception to orthodox causation and declined to recognize any broader policy-based relaxation of the requirement of proof: *AB v Ministry of Defence* [2012] UKSC 9 [75] (Lord Brown).

